# Product news

**DOI:** 10.1155/S1463924681000308

**Published:** 1981


REFERENCES
[1] The use of automation in the development and application of chromatographic methods in food analysis. Dr D. Folkes, RHM Central Research Laboratory, High Wycombe, Bucks.
[2] Automated sample preparation for HPLC-techniques, applications and some possibilities in food analysis. S. C. Coverly, Technicon Instrument Co. Ltd., Basingstoke, Hants.
[3] Applications of GC/quadrupole MS in trace food analysis, Dr. M. J. Saxby, BFMIRA, Leatherhead, Surrey.
[4] Application of near IR to the analysis of food. Dr. B.G. Osborne, Flour Milling and Baking Research Association, Chorleywood.
[5] Determinaiton of trace chloride in poultry meat using automated ion - selective electrode techniques. A.M.C. Davies, Food Research Institute, Norwich.
[6] Process instrumentation in the food factory. D.J. Steele, BFMIRA, Leatherhead, Surrey.

### Product News

### Atlantic City revisited 1981

*A review of exhibits at this year's Pittsburgh Conference*

The 32nd Annual Pittsburgh Conference on Analytical Chemistry and Applied Spectroscopy was held from 9 - 13th March at Atlantic City, NJ, USA (A report on the scientific meetings appears on page 100). In true tradition it was bigger, better attended and had more lectures presented at it than before. As a regular visitor one was left with the opinion that perhaps there is really very little new under the sun. Microprocessors were first presented several years ago and now they are mostly common place, while visual display units and graphics displays increase in numbers and sophistication. This year there was a considerable interest in computer systems and networking.

The show presented the latest state of the art instrumentation, and these instruments are more and more sophisticated – however, do they fulfill the true aims of the analyst? Very often it is sad to note that instrument companies have developed their systems without regard to the needs of the analyst; particularly they do not often allow facilities for filing data and interogation of the data at a future date.

It is impossible to cover all the aspects of instrumentation on show which related to automation, however the few which are discussed here typify those on show.

### Spectrophotometer

The new DU-5 UV-visible/NIR computing spectrophotometer displayed by Beckman automates procedures in analysing aerosols, polymers, paints, food, drugs, water and biologicals. This table-top instrument includes a spectrophotometer, microcomputer and printer in one unit with software memory storage modules. It can be programmed for specific user applications with quick change-over between analyses. The

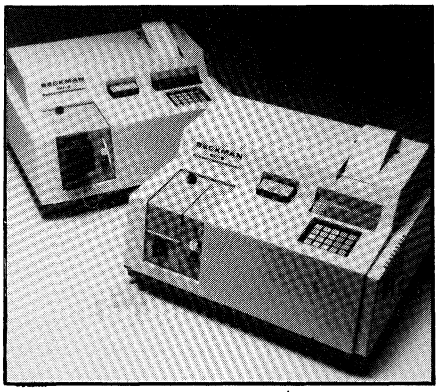

*Beckman's DU-5 UV visible/NIR computing spectrophotometer.*

DU-5 stores internally three individual analysis programs. Memory-Pac plug-in software modules offer program storage for five additional analyses with an EA-ROM non-volatile memory that will not erase in a power failure or when removed from the instrument.
*Beckman Instruments Inc., 2500 Harbor Boulevard, Box 3100, Fullerton, Cal. 92634, USA.*

### Atomic fluorescence spectrometer

The Baird Corporation introduced their Plasma/AFS which is currently the only commercially available atomic fluorescence spectrometer. Intended for the simultaneous determination of any 12 of 65 different elements, it delivers up to 1,800 determinations per hour and offers a cost-effective alternative to traditional atomic absorption and plasma emission techniques. It has a linear dynamic range of 4 - 5 orders of magnitude with "virtually no spectral interferences". Setup for a different combination of elements is fast and requires no optical or mechnical alignment.
*Baird Corporation, 125 Middlesex Turnpike, Bedford, MS 01730, USA.*

### Computer interface

UTI has developed a microprocessor-based interface which links their range of mass spectrometers with virtually any computer on the market. The Spectra-Link is compatible with the IEEE 488, RS-232C and RS-449 interface standards. The unit includes software stored in read-only memory (firmware) for five operating modes, Four of these (spectrum scan mode, total pressure mode, specific peak mode, and calibration mode) automatically perform most of the common tasks required to control the unit. The fifth, direct control mode, permits even more operator flexibility by allowing the user to write totally original programs.
*UII, 325 N. Mathilda Avenue, Sunnyvale, CA 94086, USA.*

### IR Spectrophotometers

Perkin Elmer's new product line of low cost infrared spectrophotometers, the Model 1300 series, was exhibited. There are three models; the 1310 has a single slit program and two scan speeds, the 1320 and 1330 have three scan speeds and two appropriate slit programs. The series can be interfaced to the Model 3500 infrared data station providing an improvement of spectral quality and the facility to use the spectrophotometer for 'Search' applications in the identification of unknowns.
*Perkin-Elmer Corp, Main Avenue (MS-12), Norwalk, CT 06856, USA.*

**Editor's Note:** *The address of the manufacturer/supplier appears in italics at the end of each item. In some cases this address will be that of a subsidiary to the manufacturing company as the address given is that from which the information has been obtained.*

### Surface tension measurement

Madison-Kipp demonstrated their new automatic SensaDyne 5000 surface tensiometer. Using a differential maximum bubble pressure technique the SensaDyne displays liquid surface tension valves directly in dynes/cm along with process fluid temperatures on digital displays with a resolution of 0.1 dyne/cm and 0.1°C. An optional printer provides user-programmable, continuous or intermittent records of surface tension, temperature and time. The system has been designed for research and quality assurance applications where it replaces static measurement devices. It is claimed to be the first commercial instrument for dynamic surface tension measurements.

*Madison-Kipp Corp, 201 Waubesa Street, PO Box 3037, Madison, WI 53704, USA.*

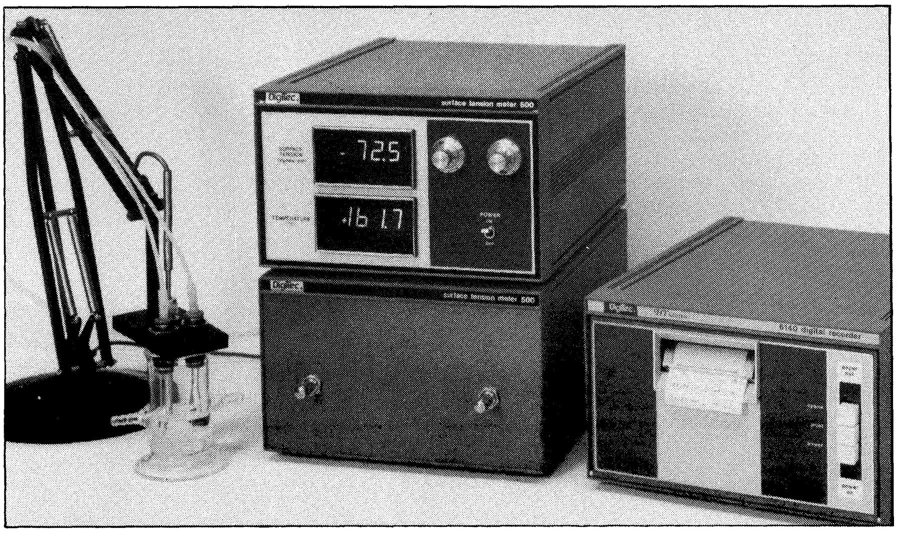

*Surface tensiometer from Madison-Kipp.*

### Liquid chromatograph

Spectra-Physics claim that their SP 8100 LC system is the world's first instrument to feature an intelligent autosampler capable of automatically reading sample identification bar codes on the vial and controlling the LC parameters without operator intervention. The SP8100 is a modular compact chromatograph specifically designed for repetitive routine analysis, small batch or methods development work. The intelligent autosampler capability allows up to 80 different samples to be loaded and analysed without operator input or intervention. The integral autosampler option, which can be retro fitted at any time, automatically reads a bar code label on each standard vial and then sets up all the instrument parameters for that sample. The operator can change or insert new samples at any time, even during repetitive batch runs, leaving the chromatograph to make all necessary adjustments and LC file selection.

*Spectra-Physics GmbH, Siemensstrasse 20, D-6100 Darmstadt, W. Germany.*

### Multi-purpose balance

The Model 5006 electronic balance from Arbor Laboratories has a capacity range of 0 - 5000 grams and a resolution readability to 0.01 grams. In addition to handling routine weighings this balance can be interfaced with complex computer or peripheral systems. A 15-position stability switch located on the back of the balance reduces operator errors. This option determines when the environment stabilises and then allows the balance to start a weighing cycle; in unstable environments the balance automatically inhibits input/output transfers which eliminates incorrect measurement recordings.

*Arbor Laboratories Inc., 3784 Fabian Way, Palo Alto, CA 94 303, USA.*

### Solution handling system

FiAtron's SHS-200 is a microprocessor controlled system with which most repetitive analytical solution handling tasks can be readily automated. It is designed to fully utilise the many advantages of flow injection analysis. In addition it allows the injection of a programmable volume of sample and reagent into the carrier stream. This 'variable sample volume mode' can readily accommodate small as well as very large sample or reagent volumes without physically changing sample loops. Additional operational modes include stop-flow, dispensing and calibration.

*FiAtron Systems Inc., 6651 N. Sidney Place, Milwaukee, WI 53209, USA.*

### Organic-free water

Millipore exhibited their new disposable cartridge for batch preparation of HPLC-grade organic-free water. Called Organex, the cartridge contains a proprietary organic scavenger resin which is said to remove virtually all trace organics from distilled or deionised water. The Organex cartridge is designed for use in an inexpensive borosilicate glass holder which can process a one litre batch of organic-free water in five to seven minutes. The holder also accepts a 0.45 membrane disc filter to remove all residual particulate matter larger than the rated pore size.

*Millipore Corp, Bedford, MA 01730, USA.*

### Automated metals analysis

A new automated multi-element metals analyser was unveiled by Labtest Equipment Company. The Labscan SAM-1 can analyse either ferrous or non-ferrous metal samples with a precision of 1-3% coefficient of variation at normal concentration ranges. It is a compact spark optical emission direct-reading spectrometer incorporating a programmable scanning monochromator with a wavelength range of 1700 - 6700A, resolution of 0.6A first order and a focal lengrh of 350 mm. It operates under the control of a self-contained computer with an 8085 control processing unit, 4K PROM, 8K RAM and a 100 hour battery backup system.

*Labtest Equipment Company, 11828 LA Grange Avenue, Los Angeles, CA 90025, USA.*

### Thermometric titrator

The Sanda Thermo-Titrator system provides an enthalpimetric method using temperature change to indicate the course of a particular reaction between analyte and reagent. Thermometric titrations are free energy and enthalpy dependent measurements. Free energy measurements are generally logarithmic, ie, the stoichiometric equivalence point must be approached alowly. In contrast thermometric measurements are linear and the endpoint may be approached at a constant speed over a time interval of 30 seconds or less.

*Sands Inc., 4343 East River Drive, Philadelphia, Pa 19129, USA.*

### Thermal analysis

A British newcomer to the show was Stanton Redcroft who displayed an extensive range of thermal analysis test equipment. Their STA 780 simultaneous thermobalance differential thermal analyser uses microprocessors to allow the rate of change of weight and thermal characteristics of many different materials to be studied simultaneously.

*Stanton Redcroft Ltd, Copper Mill Lane, London, SW17 OBN. UK.*

### Flow injection analysis

American Research Products unveiled their automated multiple flow injection analysis system, the AMFIA-2000. Using a non-segmented continuous flow principal, the microprocessor controlled system features the ability to analyse 150-360 samples per hour. It has easily changed chemistry modules and uses peak area integration of calculations. Mathematics programs permit the selection of 3 to 10 standards (including water) as well as a minimum coefficient of correlation acceptable in using a least squares fit routine for linear or non-linear chemical analysis. The system includes a newly designed 200-position auto sampler and colorimeter with transmission or absorbance readout, accuracy of 0.01 units, and up to four selectable filters for wavelength setting. A new fluorescence detector with digital readout and a BCD output is also available.
*American Research Products Inc., 4232A Howard Ave., Kensington Md 20795, USA.*

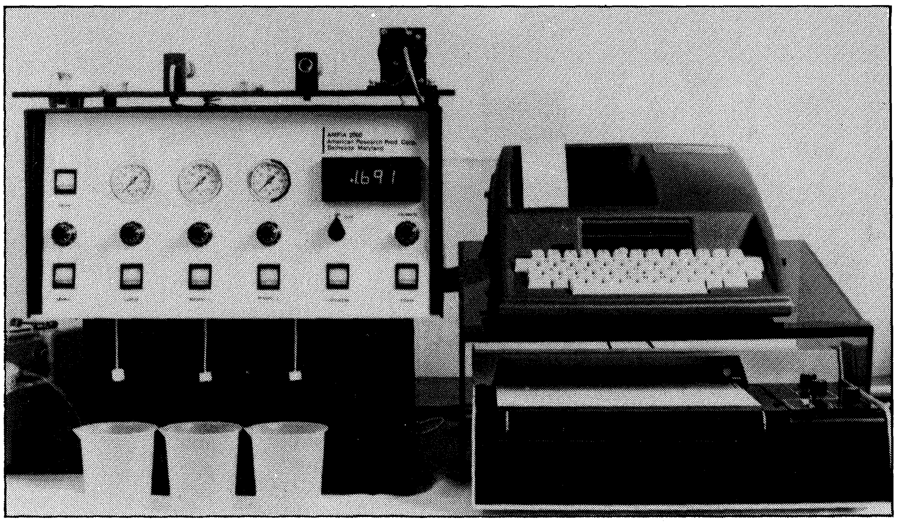

*Multiple flow injection analysis system from American Research Products.*

### Titration system

Fisher Scientific chose the Pittsburgh conference to introduce their Titrimeter II AEP system. This is a semi-automatic single-sample titrating system which, because it can operate in both fixed-endpoint and endpoint-seeking modes, can handle aqueous and non-aqueous samples. The Titrimeter II operates over a 1-1000 mV or 0-14 pH range, incorporating microprocessor control to automatically deliver titant with an accuracy of 1.1% of syringe volume and 1.2% precision. Titrant volume is continuously displayed on an LED readout.
*Fisher Scientific Co. 711 Forbes Avenue, Pittsburgh. PA 15219. USA.*

### Atomic absorption spectrometer

Two new atomic absorption spectrometers were shown by Varian Associates, with special software and a programmable sample changer to provide a completely automated system for metals analysis. The AA 1275/1475 series has an 8-bit microcomputer built-in for data collection, system management and direct read-out of metal concentrations. The AA 1275 is a single beam instrument; the AA 1475 employs a time shared double beam system for source drift compensation. Both instruments have flame emission capabilities.
*Varian Associates, 611 Hansen Way, Palo Alto. CA 94303, USA.*

